# Numerical Investigation on the Effects of Dielectric Barrier on a Nanosecond Pulsed Surface Dielectric Barrier Discharge

**DOI:** 10.3390/molecules24213933

**Published:** 2019-10-31

**Authors:** Shen Zhang, Zhenli Chen, Binqian Zhang, Yingchun Chen

**Affiliations:** 1 School of Aeronautics, Northwestern Polytechnical University, Xi’an 710072, China; shenzhang@mail.nwpu.edu.cn (S.Z.); bqzhang@nwpu.edu.cn (B.Z.); 2 Commercial Aircraft Corporation of China, Ltd., Shanghai 200120, China; chenyingchun@comac.cc

**Keywords:** nanosecond pulsed dielectric barrier discharge, dielectric constant, dielectric barrier thickness, surface charge, streamer characteristics, gas heating

## Abstract

In order to understand the impacts of dielectric barrier on the discharge characteristics of a nanosecond pulsed surface dielectric barrier discharge (NS-DBD), the effects of dielectric constant and dielectric barrier thickness are numerically investigated by using a three-equation drift–diffusion model with a 4-species 4-reaction air chemistry. When the dielectric constant increases, while the dielectric barrier thickness is fixed, the streamer propagation speed (*V*), the maximum streamer length (*L*), the discharge energy ($Q_{D\_ei}$), and the gas heating ($Q_{GH}$) of a pulse increase, but the plasma sheath thickness (*h*), the fast gas heating efficiency η, and the charge densities on the wall surface decrease. When the dielectric barrier thickness increases, while the dielectric constant is fixed, *V*, *L*, $Q_{D\_ei}$, and $Q_{GH}$ of a pulse decrease, but *h*, η, and the charge densities on the wall surface increase. It can be concluded that the increase of the dielectric constant or the decrease of the dielectric barrier thickness results in the increase of the capacitance of the dielectric barrier, which enhances the discharge intensity. Increasing the dielectric constant and thinning the dielectric barrier layer improve the performance of the NS-DBD actuators.

## 1. Introduction

The nanosecond pulsed dielectric barrier discharge (NS-DBD) in high pressure air produces non-equilibrium plasma. The different degrees of freedom of the electrons, ions, and neutrals in the plasma are not in equilibrium among each other, so that the high energy electrons and the radical energetic species are produced. The electrohydrodynamic, gas heating, light emission, and the plasma–surface interactions occur in the plasma. Therefore, the NS-DBD actuators can be applied in plasma etching [1], coating [2], biology medicine [3], gas conversion [4], aerodynamics [5] and combustion [6]. In particular, the volatile organic compounds (VOCs) degradation by the DBD plasma actuators has been intensively investigated, and it has shown that the method has potential application in air-cleaning systems for the benzene removal [7], toluene, and xylene destruction [8,9,10]. The plasma–surface interactions, such as the emission of secondary electrons and the charging of the electrons and ions, are very crucial to the etching, coating, and biology medicine applications. The gas heating of the NS-DBD actuators are very important in the aerodynamics and combustion applications.

The performance of the NS-DBD actuator is mainly determined by the applied voltage, the size, the arrangement, and the materials of the electrodes and the dielectric barrier. Among them, the parameters of the dielectric barrier are very crucial, they affect the plasma–surface interactions and the gas heating of the actuators. There are few investigations on the effects of dielectric barrier. The dielectric constant and the dielectric barrier thickness are the two dominant parameters that affect the discharge characteristics; they have been studied in [11,12,13,14] and [11,13,15,16], respectively. It was found that the current and the surface charge density on the dielectric barrier surface increase with the dielectric constant. The streamer thickness increases with the dielectric constant and the dielectric barrier thickness, but the maximum streamer length decreases with the dielectric constant. The total discharge energy and the gas heating increase with the dielectric constant, but they decrease with the dielectric barrier thickness.

In these previous experiments, the effects of dielectric barrier on the gas heating and the charge distributions on the wall surface are difficult to be analyzed in detail, due to the limitation of the experimental methods. The gas heating are usually deduced approximately either quantitatively from the temperature increment of the gas in the discharge volume or qualitatively from the size of the heating area in the Schlieren images [13]. The charge distributions on the wall surface can hardly be estimated. Additionally, the mechanism of the effects of dielectric barrier cannot be analyzed in the experiments. Therefore, the numerical method should be adopted to study the effects of the dielectric barrier.

The computational cost of the simulations of the NS-DBD discharges by using kinetic models at high pressure is formidable [17,18]. Because the gas is weakly ionized at high pressure in the NS-DBD, it can be reasonably assumed that only the electron-neutral, ion-neutral collisions can occur, whereas the magneto-hydrodynamic effects can be neglected [19]. Therefore, the fluid models based on the drift–diffusion approximations can be an alternative compromising the computational cost and fidelity [20,21,22].

Although the previous studies have partly studied the effects of dielectric barrier on the discharge characteristics, there is no systematic quantitative investigation on the plasma–surface interactions and gas heating of the NS-DBD plasma actuators. The objective of the present work is to investigate the effects of the dielectric constant and the dielectric barrier thickness on the plasma–surface interactions and thermal effects of an asymmetric assembled NS-DBD actuator. The dielectric materials are fluorocarbon, impregnated paper, Kent wax and glass, with the dielectric constant increasing from 2.7 to 10.0. Although the impregnated paper and Kent wax have not been used as the dielectric barrier material, the numerical investigation in the present work gives an quantitative study on them. The dielectric barrier thickness is changed from 0.15 mm to 0.9 mm. The discharge is modeled by using a three-equation drift–diffusion fluid model. In the air chemistry, four species, i.e., electrons, positive ions, negative ions, and neutral species, and four kinds of reactions among them, are chosen to reduce the computational cost. Local-mean-energy approximation (LMEA) is used to obtain the reaction-rate coefficients of the electron–impact reactions and the electron–transport coefficients. The streamer morphology, total discharge energy, gas heating and the corresponding efficiency, and the transportations and distributions of the electrons, ions, and charge on the wall surface are studied.

## 2. Models and Numerical Methods

The physical model and the numerical methods are the same as the ones in the previous paper [23].

### 2.1. Physical Model

#### 2.1.1. Discharge Model

The discharge is modeled by a three-equation drift–diffusion fluid model, which contains the mass-conservation equations, an energy-conservation equation for the electrons and Poisson’s equation for the pulse. The mass and energy conservation equations of the electrons are

(1) $$\frac{\partial }{\partial t}\left(n_{e}\right)+\nabla \cdot {\vec{\Gamma }}_{e}=R_{e},$$

(2) $$\frac{\partial }{\partial t}\left(n_{\varepsilon }\right)+\nabla \cdot {\vec{\Gamma }}_{\varepsilon }+\vec{E}\cdot {\vec{\Gamma }}_{e}=R_{\varepsilon },$$

where the subscripts *e* and ε indicate electron and electron energy, respectively. $n_{e}$ and $n_{\varepsilon }$ are the electron–number density and the electron–energy density. $R_{e}$ and $R_{\varepsilon }$ are the sources of the electrons and the electron energy due to chemical reactions. ${\vec{\Gamma }}_{e}$ and ${\vec{\Gamma }}_{\varepsilon }$ are the fluxes of the electron and the electron energy, which are

(3) $${\vec{\Gamma }}_{e}=-\left(\mu_{e}\vec{E}\right)n_{e}-\nabla \left(D_{e}n_{e}\right)+n_{e}{\vec{v}}_{c},$$

(4) $${\vec{\Gamma }}_{\varepsilon }=-\left(\mu_{\varepsilon }\vec{E}\right)n_{\varepsilon }-\nabla \left(D_{\varepsilon }n_{\varepsilon }\right)+n_{\varepsilon }{\vec{v}}_{c},$$

in the drift–diffusion approximation. The drift and diffusion coefficients of the electron and the electron energy are calculated by a Boltzmann equation solver Bolsig+ [24] through the electron energy probability function (EEPF) $f_{0}$ [25],

(5) $$\mu_{e}=-\frac{\gamma }{3N}\int_{0}^{\infty }\frac{\varepsilon }{\sigma_{m}}\left(\frac{\partial f_{0}}{\partial \bar{\varepsilon }}\right)d\varepsilon ,$$

(6) $$D_{e}=\frac{\gamma }{3N}\int_{0}^{\infty }\frac{\varepsilon }{\sigma_{m}}f_{0}d\varepsilon ,$$

(7) $$\mu_{\varepsilon }=-\frac{\gamma }{3N\bar{\varepsilon }}\int_{0}^{\infty }\frac{\varepsilon^{2}}{\sigma_{m}}\left(\frac{\partial f_{0}}{\partial \bar{\varepsilon }}\right)d\varepsilon ,$$

(8) $$D_{\varepsilon }=\frac{\gamma }{3N\bar{\varepsilon }}\int_{0}^{\infty }\frac{\varepsilon^{2}}{\sigma_{m}}f_{0}d\varepsilon ,$$

where *N* is the gas number density and $m_{e}$ ($m_{e}$ is the mass of an electron) is a constant. $\sigma_{m}$ is the effective momentum transfer cross section. ${\vec{v}}_{c}$ is the gas velocity. The source terms $R_{e}$ and $R_{\varepsilon }$ are $\sum \left(\left(\alpha -\eta \right)\left|{\overset{⇀}{\;\Gamma }}_{e}\right|+k_{r}\Pi n_{r}\right)$ and $\sum \left(\left(\alpha -\eta \right)\left|{\overset{⇀}{\;\Gamma }}_{\varepsilon }\right|\Delta \varepsilon +k_{r}\Delta r\Pi n_{r}\right)$, where α is the ionization coefficient and η is the attachment coefficient. $k_{r}$ is the reaction rate and $n_{r}$ is the number density of the reactants. Δε and Δr are the energy loss of electronic ionization reactions and other electron impact reactions.

The mass-conservation equations of the heavy species are

(9) $$\rho \frac{\partial }{\partial t}\left(\omega_{k}\right)=\nabla \cdot {\vec{\Gamma }}_{k}+R_{k},$$

where $\omega_{k}$ is the mass fraction of the *k* heavy species. $R_{k}$ is the source term of the species *k* due to the chemical reactions. ${\vec{\Gamma }}_{k}$ are the drift–diffusion approximation fluxes

(10) $${\vec{\Gamma }}_{k}=\rho \omega_{k}\left(D_{k}\frac{\nabla \omega_{k}}{\omega_{k}}+D_{k}\frac{\nabla M}{M}-z_{k}\mu_{k}\vec{E}\right)+\rho \left(\omega_{k}{\vec{v}}_{c}\right),$$

where $\mu_{k}$ and $D_{k}$ denote the drift and diffusion coefficients of the *k* heavy species, respectively. The drift coefficients of ions are the functions of the reduced electric field [26]. The diffusion coefficients of ions and neutrals are calculated from the generalized Einstein relation and the classical gas kinetic theory [27,28], respectively. $z_{k}$ is the charge number. *M* is the average molar mass. ρ is the gas density, which is calculated by the equation of state for perfect gas as

(11) $$\rho =\frac{pM}{RT},$$

where *p* and *T* are the gas pressure and temperature, respectively. *R* is the universal gas constant.

The electric potential ϕ induced by the space net charge is governed by the Poisson’s equation

(12) $$-\nabla \cdot \left(\varepsilon \nabla \phi \right)=e\sum \left(-n_{e}+z_{k}n_{k}\right),$$

where *e* is the elementary charge and ε is the permittivity. ε is 1.0 in the air and a dielectric constant larger than 1.0 in the dielectric barrier, respectively. $n_{k}$ are the number densities of the heavy species and nk=ωkNAρωk·NA·ρMkMk, where $N_{A}$ is the Avogadro constant. Electric field is calculated from the potential as $\vec{E}=-\nabla \phi$.

#### 2.1.2. Discharge Energy and Gas Heating

The discharge energy is determined by the currents of the electrons, ions, and the intensity of the electric field. It is calculated by integral in the computational domain and discharge time as

(13) $$\begin{array}{cc} Q_{D\_ei}= & \int_{0}^{T_{p}}\;\int \int \;P_{D\_ei}dxdydt=\int_{0}^{T_{p}}\;\int \int \;\left(P_{D\_e}+P_{D\_i}\right)dxdydt=\int_{0}^{T_{p}}\;\int \int \;\left({\vec{J}}_{e}\cdot \vec{E}+{\vec{J}}_{i}\cdot \vec{E}\right)dxdydt\\ = & Q_{D\_e}Q_{D\_i}, \end{array}$$

where $T_{p}$ is the integrating time. D_ei, D_e, and D_i denote the discharge energy, the electrons’ energy, and the ions’ energy.

The gas heating is composed of the energy of electron elastic-collision and rotational excitation (el−r), the electron energy spent in electronic excitation (*E*) [29], the energy stored in the vibrational excitation (*V*) and the ions’ energy [23]. The energy and the efficiency of the gas heating(GH) are

(14) $$Q_{GH}=\int_{0}^{T_{p}}\left(P_{GH}\right)dt\;=\int_{0}^{T_{p}}\left(P_{D\_i}+P_{el-r}+P_{E}+P_{VT}\right)dt\;,$$

(15) $$\eta_{GH}=Q_{GH}/Q_{D\_ei}=P_{GH}/P_{D\_ei}.$$

#### 2.1.3. Air Chemistry

The air chemistry contains a positive ion $Air^{+}$, a negative ion $Air^{-}$, the electron *e*, and a ground state of neutral heavy particle Air. Air denotes the average of the $N_{2}$ and $O_{2}$ molecules, and the molar fraction is 4:1. The air–chemistry model incorporates 4-species and 4-reaction mechanisms, as shown in Table 1, which is the same as that in the previous paper [23].

### 2.2. Numerical Method

The governing Equations (1), (2), (9) and (12) are solved by using a finite element method [30,31]. A second-order Lagrange shape function is used for each element. Because the magnitudes of the variables, such as potential, electron–number density, ions–number density and neutrals–number density, are very different, the dependent variables are non-dimensionalized to the magnitude of 1 in each step to avoid ill-conditioned problems. A second-order implicit backward differentiation formula (BDF) [32,33] method is used for the time integration. The application of the BDF method to the discretized partial differential equation system results in a nonlinear algebraic system at each time step. The nonlinear algebraic system is solved by an affine invariant form of the damped Newton method [34]. This leads to a linear equation system for each Newton correction. The linear equation system is solved by a direct multi-frontal massively parallel sparse method (MUMPS) [35]. The MUMPS works on general systems of the form Ax=b. It uses nested dissection pre-ordering algorithms to permute the columns and thereby minimizes the fill-in.

The initial conditions, the boundary conditions, and the time steps of the models are the same as the ones in the previous paper [23]. The effects of the secondary emission are considered in the present work. It is assumed that the ions’ contact with the solid wall and return to the plasma to be their parent neutral molecules. The positive ions induce secondary electron emission through Auger neutralization process [30]. Baragiola et al. [36] found that the induced electron flux is proportional to the positive ions’ flux with a secondary electron emission coefficient $\gamma_{p}\approx 0.016\times \left(E^{th}-2\times \varphi \right)$, where $E^{th}$ and φ are the ionization potential of the impacting ion and the material surface work function, respectively. $\gamma_{p}$ of the boundaries of the copper electrode is 0.02. $\gamma_{p}$ of the boundaries of fluorocarbon, impregnated paper, Kent wax and glass are 0.05, 0.06, 0.07, and 0.08 [37], respectively. Because the emitted electrons are expected to gain an amount of energy that greatly exceeds the assumed initial value within the adjacent sheath region, the average secondary electron emission energy ${\bar{\varepsilon }}_{a}$ is assumed to be 1 eV [38].

The physical models and the numerical method in this work are validated by two benchmark cases [23]. The first case is a two-dimensional simulation of an NS-DBD [39], and the second case is an experiment of an asymmetric assembled NS-DBD [40].

### 2.3. Simulation Setup

The NS-DBD actuator is set to be the same as that in the experiment of Starikovskii et al. [40]. The actuator consists of an exposed electrode and a grounded electrode. Both electrodes are made of copper foil having a thickness of 0.05 mm and a width of 5 mm. The two electrodes are separated by a dielectric barrier layer having different dielectric constants and different thickness in different cases, as shown in Table 2. The computational domain has a length of 80 mm and a height of 30 mm; the geometry and the mesh distributions are the same as the ones in the paper [23]. The initial number densities of the electrons and the negative ions are both $2.5\times 10^{11}\;m^{-3}$. The number density of positive ions is $5\times 10^{11}\;m^{-3}$. The mole fraction of Air is calculated based on the principle of mass conservation. The pressure and the temperature are 64 kPa and 259K, corresponding to an altitude of 4.4 km in the standard atmosphere.

**Figure 1:**
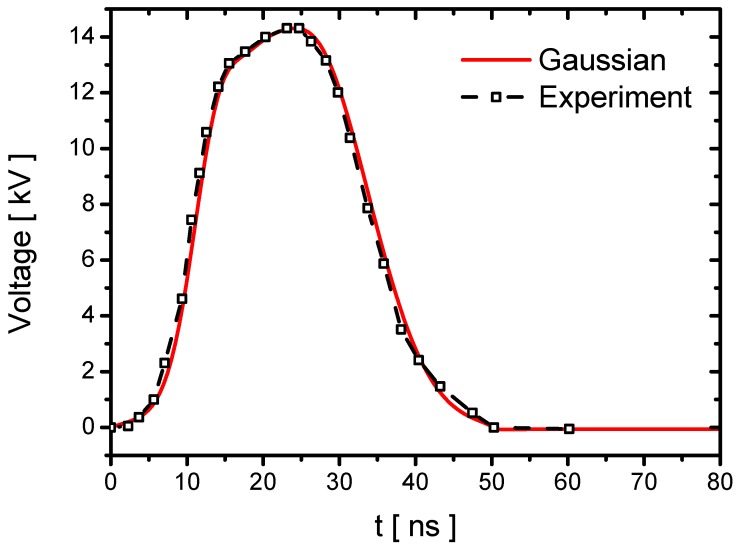
Waveforms of the experiment voltage and the fitted Gaussian voltage. Peak voltage $U_{peak}=14$ kV, rising time $\tau_{rise}=25$ ns and pulse duration $\tau_{pulse}=50$ ns.

A positive pulse waveform is applied to the actuator with a peak $U_{peak}$ of about 14 kV. The rising time $\tau_{rise}$ is 25 ns. The pulse duration $\tau_{pulse}$ is 50 ns. The experimental waveform is fitted by a Gaussian function, as shown in Figure 1.

The effects of dielectric constant (ε) and thickness ($t_{d}$) of the dielectric barrier on the discharge characteristics are investigated from Case 1 to Case 7, which are summarized in Table 2.

Case 1 is the base case, where the dielectric material is fluorocarbon with ε=2.7, and $t_{d}=0.3$ mm. The dielectric material in Case 2, Case 3, and Case 4 are impregnated paper, Kent wax, and glass, respectively. The effects of dielectric constant are studied in the first four cases, where $t_{d}$ is kept constant. The effects of dielectric barrier layer thickness are investigated by the comparison of the base case with the last three cases, where ε keeps constant.

## 3. Effects of Dielectric Barrier

In practical applications, the dielectric barrier can be changed to optimize the performance of the NS-DBD actuators. The dielectric material, thickness, length, surface roughness, and arrangement of the dielectric barrier vary differently. According to the previous studies, the dielectric material and thickness have strong effects on the discharge characteristics. The main properties of dielectric materials are dielectric constant, thermal conductivity, electric volume resistivity, and specific heat. In the present work, the effects of dielectric materials are studied by changing the dielectric constant, as in the previous studies [11,14]. As a result, the effects of the dielectric constant (ε) and dielectric barrier thickness ($t_{d}$) are investigated in the following.

### 3.1. Effects of Dielectric Constant

The effects of dielectric constant on the electric characteristics, the streamer morphology, and discharge energy have been studied experimentally and numerically in the past studies [11,13,14,41]. However, the effects on the electrical characteristics, surface charge, streamer morphology, discharge energy, and gas heating have not been investigated systematically. Here, the effects of ε are investigated.

#### 3.1.1. Plasma Characteristics and Current

**Figure 2:**
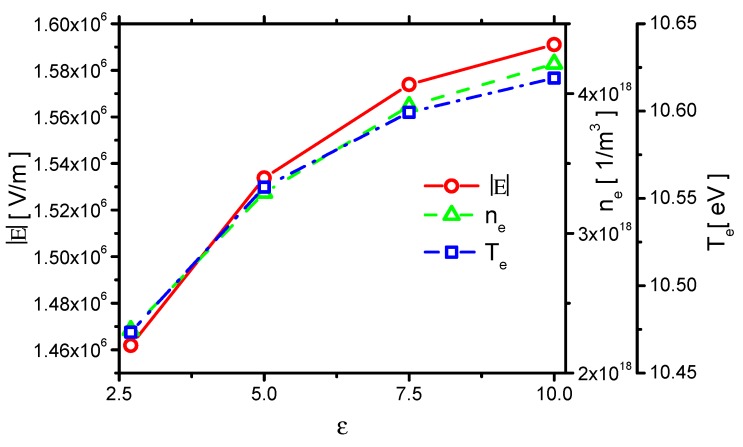
Effects of the dielectric constant ε on the electric field strength $\left|\overset{⇀}{\;E}\right|$, electron density $n_{e}$, and electron temperature $T_{e}$.

Because the electric strength, electron temperature, and the electron density are the key plasma parameters, the effects of ε on $\left|\overset{⇀}{\;E}\right|$, $n_{e}$, and $T_{e}$ are summarized and shown in Figure 2. The values are averaged in time over one pulse, and, in space, over the streamer area, as the ones in the past similar study [42]. $\left|\overset{⇀}{\;E}\right|$ increases with increasing ε. This is because a higher dielectric constant leads to stronger polarization of the dielectric material; with more charge inside the dielectric barrier, the capacitance also increases, a greater voltage difference between the dielectric and the exposed electrode is obtained. The electric field strength increases. As a result, the electron density and electron temperature also increase with increasing dielectric constant.

**Figure 3:**
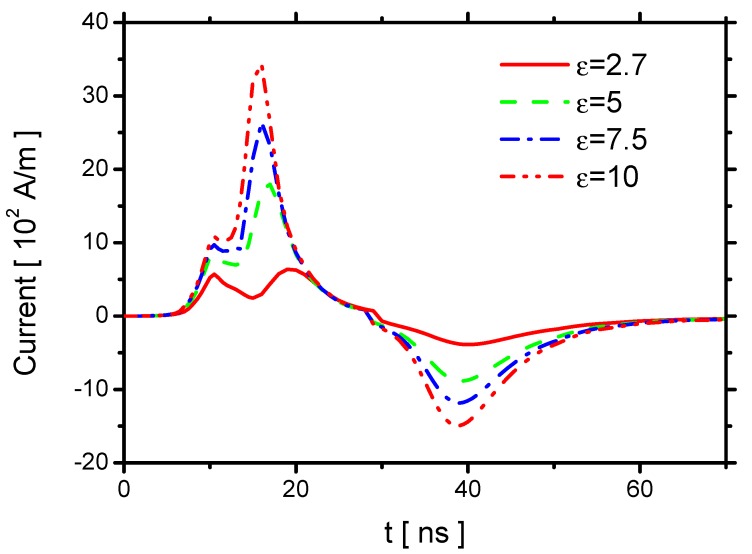
Effects of ε on the current.

The effects of ε on the currents are shown in Figure 3. The current in the experiment has not been given, Therefore, there is no direct comparison between the results of simulation and the experiment. In the previous studies, the simulated currents were compared with experimental [43] and simulation results [23] in detail for different voltage pulses, which validated the present methods. In the voltage-rise phase, the discharge occurs and extinguishes before the applied voltage reaches its peak. The currents have two spikes, and the two spikes are also observed in previous NS-DBD discharges [44]. The first spike is due to the wave ionization in the streamer propagation and displacement current, which increase with increasing ε. The second spike is caused by the repeated re-ignition on the plasma area covered previously by the streamer, which is mainly determined by the capacitance of the dielectric barrier [41,44], and it also increases with increasing ε. After the second spike, the current decreases and then increases reversely during the voltage-fall phase due to the ignition of a reverse discharge. The currents in the reverse discharge in the fall phase also increase with increasing ε because of the increase of the electric field strength.

According to a past numerical investigation [11], the electric current increases from 50–100 A/cm to 150–200 A/cm, when ε increases from 16 to 80. In another experiment investigation [12], the current also increases when the dielectric material is changed from glass (ε=5) to ceramics (ε=1000). In addition, the average electric field, electron density and electron temperature increase with ε increases from 5 to 25 in a similar experimental study on packed bed plasma reactors [42]. Therefore, the effects of ε on the plasma parameters and current in the present study are the same to the ones in the past investigations.

#### 3.1.2. Surface Charge

**Figure 4:**
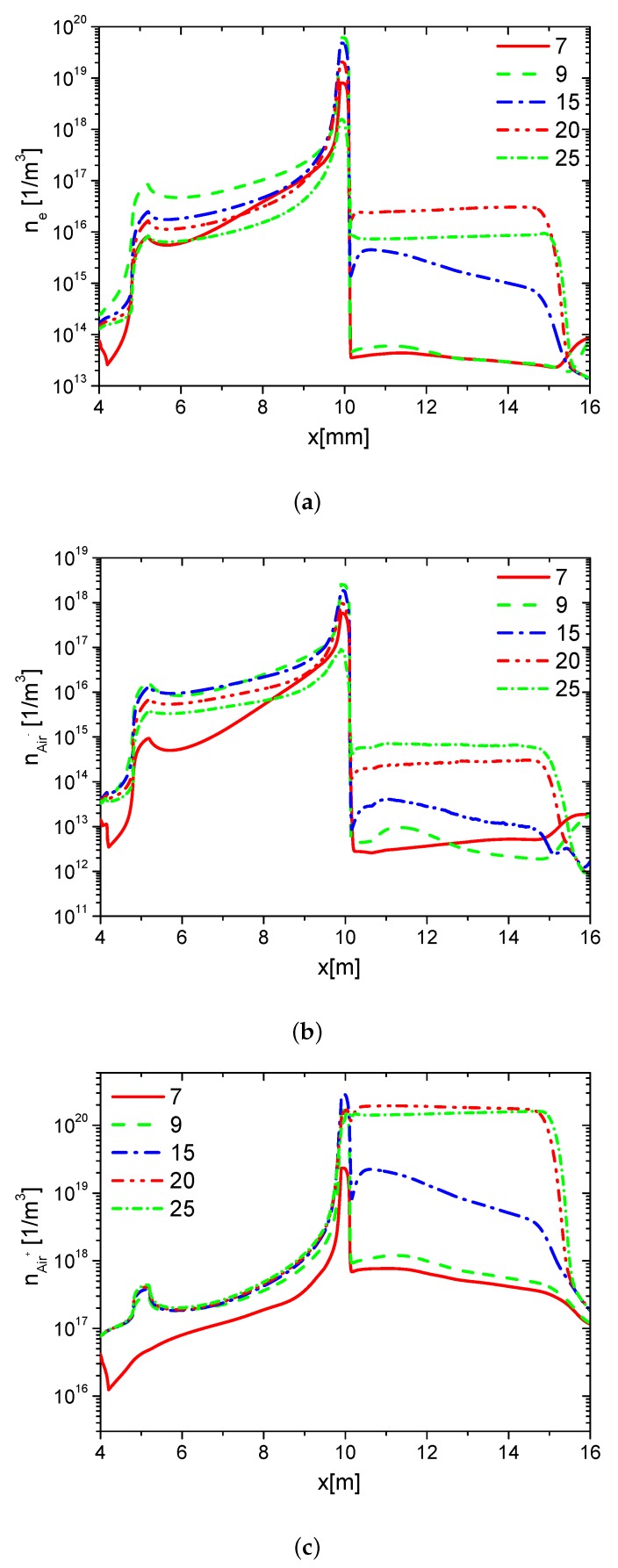
The distributions of the densities of electrons ($n_{e}$), negative ions ($n_{Air^{-}}$) and positive ions ($n_{Air^{+}}$) on the wall surface at time 7 ns, 9 ns, 15 ns, 20 ns, and 25 ns in the rise phase in Case 1.

First, to investigate the effects of ε on the charge on the wall surface, the distributions of the densities of electrons, ions, and the charge in the base case (Case 1) are analyzed. The distributions of $n_{e}$, $n_{Air^{-}}$, and $n_{Air^{+}}$ at different times in the rise phase are shown in Figure 4a–c, respectively. Due to the ionization in the streamer and the charge accumulation on the dielectric surface, $n_{e}$, $n_{Air^{-}}$, and $n_{Air^{+}}$ increase on the dielectric surface (from 10 mm to 15 mm). In the rise phase of the forward discharge, the direction of the horizontal electric field points from the exposed electrode to the dielectric surface. Because $Air^{+}$ drifts along the electric field, but the electrons and $Air^{-}$ drift against the electric field, $n_{Air^{+}}$ on the dielectric surface is higher than the one on the surface of the exposed electrode (from 5 mm to 10 mm). However, $n_{e}$ and $n_{Air^{-}}$ on the dielectric surface are lower than the ones on the surface of the exposed electrode. Because there is nearly no charge accumulation on surface of the metal electrode, $n_{e}$, $n_{Air^{-}}$, and $n_{Air^{+}}$ change little on the surface of the exposed electrode.

**Figure 5:**
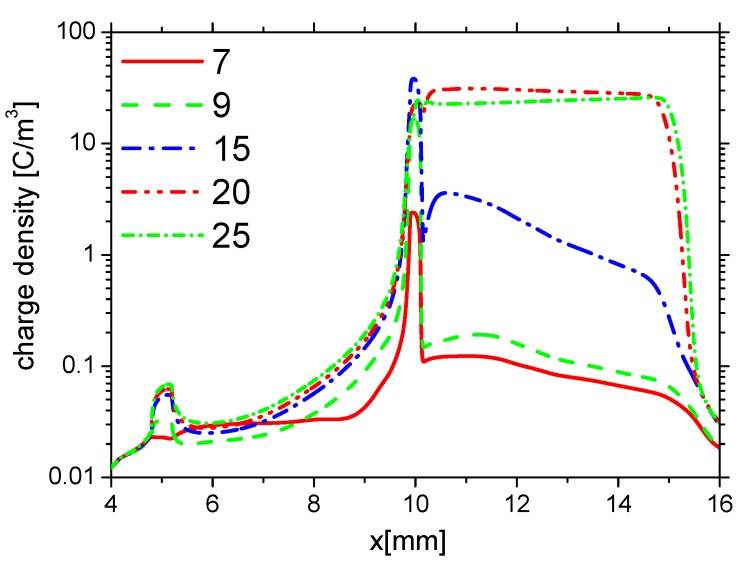
The distributions of charge density on the wall at time 7 ns, 9 ns, 15 ns, 20 ns, and 25 ns in the rise phase in Case 1.

The distributions of the densities of charge on the wall surface at different times in the rise phase are shown in Figure 5. In the rise phase of the forward discharge, the charge positively accumulates from the edge of the exposed electrode to the dielectric surface following the streamer development. At the end of the rise phase 25 ns, the charge accumulates over the entire dielectric surface, and the length of the charge is nearly equal to the length of the grounded electrode.

**Figure 6:**
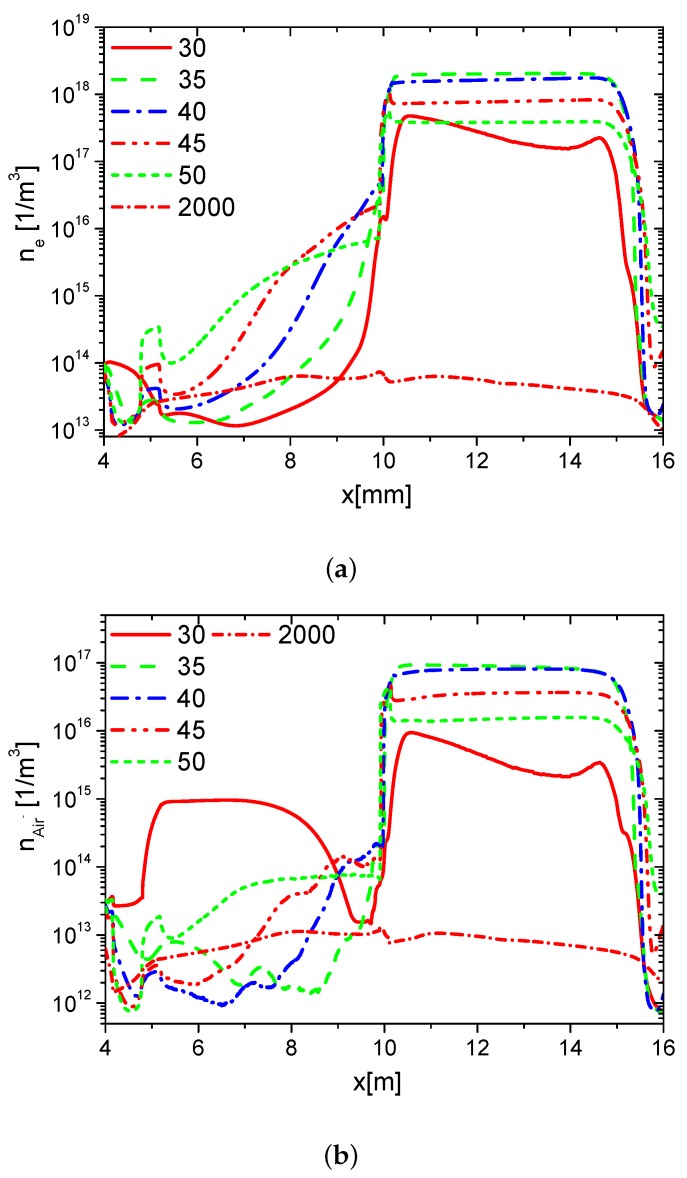

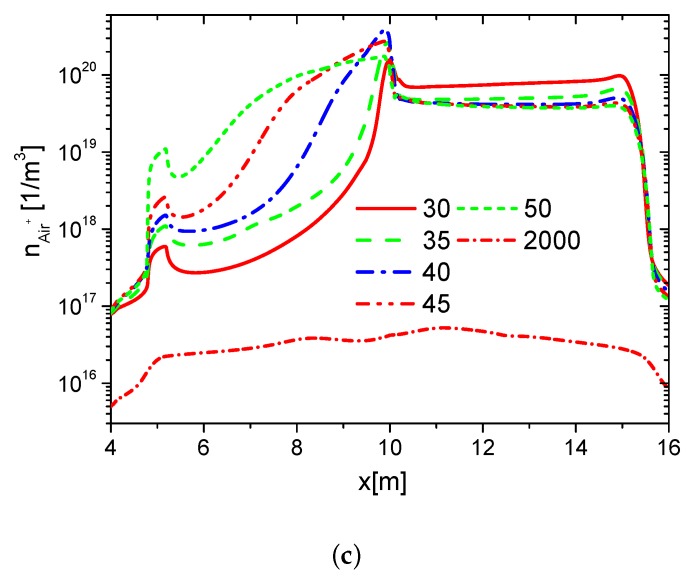
The distributions of $n_{e}$, $n_{Air^{-}}$ and $n_{Air^{+}}$ on the wall surface at time 30 ns, 35 ns, 40 ns, 45 ns, 50 ns, and 2000 ns in the fall phase in Case 1.

**Figure 7:**
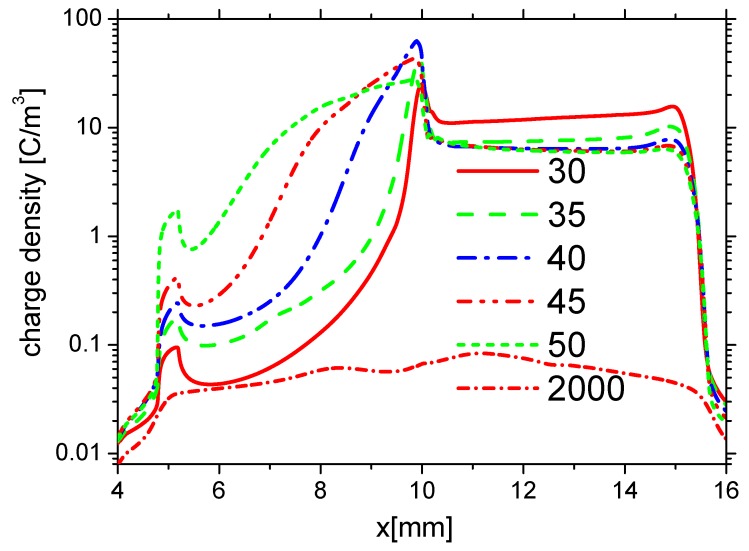
The distributions of charge density on the wall at time 30 ns, 35 ns, 40 ns, 45 ns, 50 ns, and 2000 ns in the fall phase in Case 1.

The distributions of densities of $n_{e}$, $n_{Air^{-}}$ and $n_{Air^{+}}$ at different time in the fall phase are shown in Figure 6a–c, respectively. Because the direction of the horizontal electric field is opposite to the one in the rise phase, $n_{e}$ and $n_{Air^{-}}$ on the surface of the dielectric barrier are mainly larger than the ones on the exposed electrode surface. However, $n_{Air^{+}}$ on the dielectric surface is lower than the one on the right edge of the exposed electrode surface. Because the streamer develops mainly on the edge of the surface of the the exposed electrode, $n_{e}$, $n_{Air^{-}}$, and $n_{Air^{+}}$ increase on the edge of the exposed electrode surface following the streamer development. After the pulse, the densities of the electrons and ions decrease, $n_{e}$, $n_{Air^{-}}$, and $n_{Air^{+}}$ are almost the same on the surfaces of the dielectric and the exposed electrode. The distributions of the densities of charge on the wall surface at different times in the fall phase are shown in Figure 7. In the fall phase, the charge transfers from the surface of the dielectric barrier to the exposed surface. In addition, the charge decays in almost 2000 ns.

**Figure 8:**
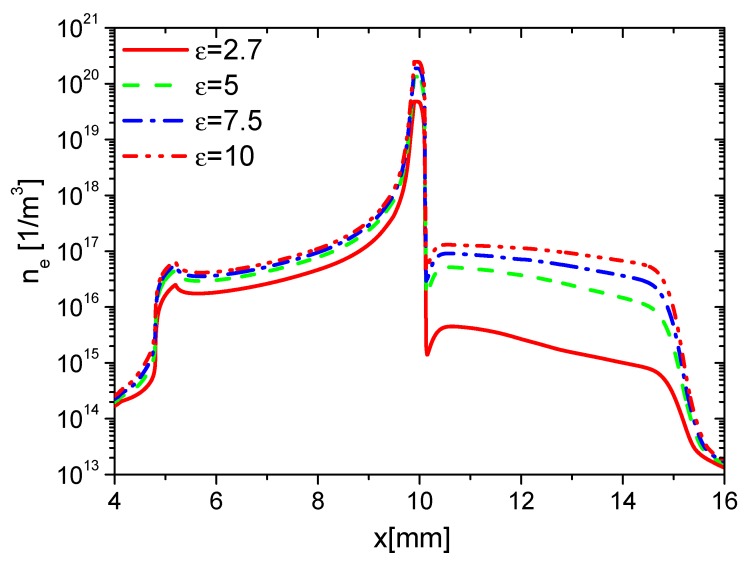
Effect of ε on the distributions of $n_{e}$ at 15 ns in the rise phase.

**Figure 9:**
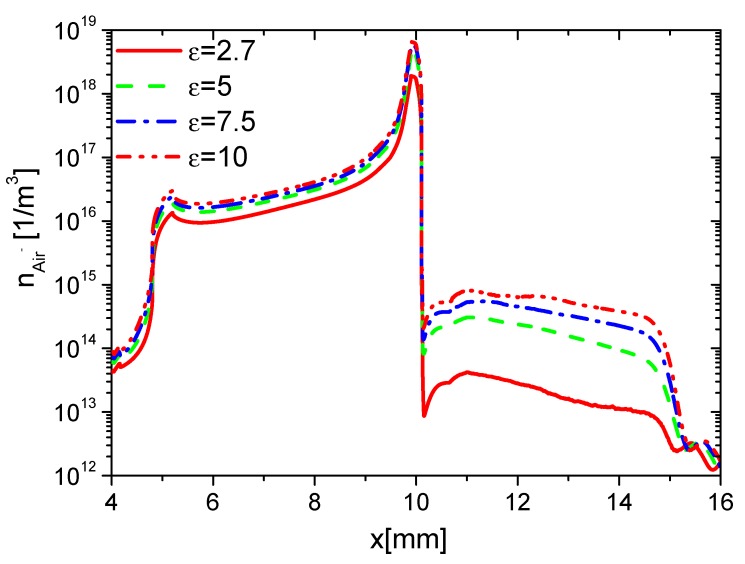
Effect of ε on the distributions of $n_{Air^{-}}$ at 15 ns in the rise phase.

**Figure 10:**
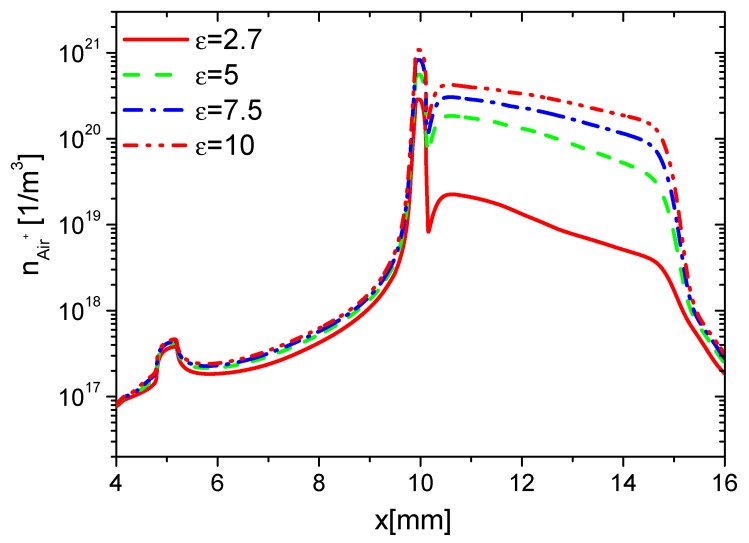
Effect of ε on the distributions of $n_{Air^{+}}$ at 15 ns in the rise phase.

Second, the effects of ε on the charge on the wall surface, the densities of electrons, and ions are analyzed. In the rise phase at 15 ns, the distributions of $n_{e}$ in Case 1 to Case 4 are shown in Figure 8. Because the average $\left|\overset{⇀}{\;E}\right|$ and $n_{e}$ increases with increasing ε, $n_{e}$ on the surfaces of the dielectric barrier and exposed electrode increases with increasing ε. However, the increment of $n_{e}$ on the dielectric surface is higher than the one on the exposed electrode due to the surface charge accumulation. The densities of $n_{Air^{-}}$ and $n_{Air^{+}}$ at 15 ns are shown in Figure 9 and Figure 10, respectively. For the same reason, $n_{Air^{-}}$ and $n_{Air^{+}}$ also increase with increasing ε, which is similar to the ones of $n_{e}$.

**Figure 11:**
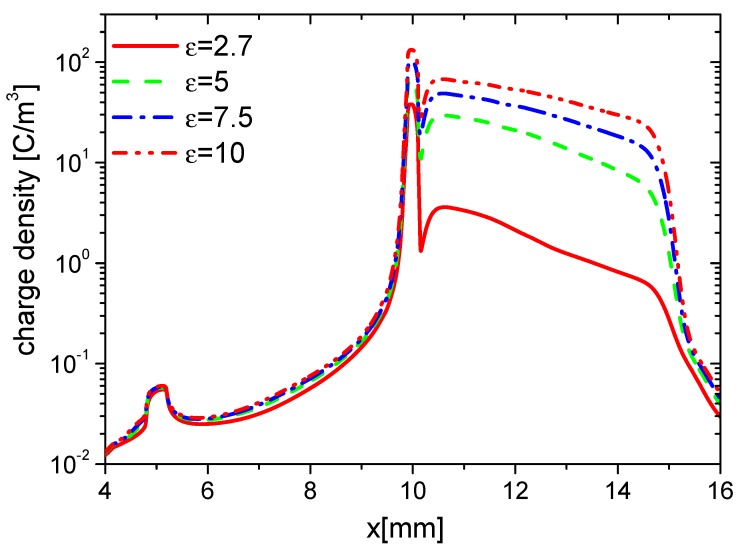
Effect of ε on the distributions of the wall charge density at 15 ns in the rise phase.

The effects of ε on the distributions of wall charge density at 15 ns are consequently studied and shown in Figure 11. Because of the increasing $\left|\overset{⇀}{\;E}\right|$, the densities of electrons and ions increase with increasing ε, and the charge accumulation on the dielectric surface is enhanced. In addition, the dielectric is stronger polarized due to higher ε, more charge is induced inside and on the surface of the dielectric barrier. However, the charge on the surface to the exposed electrode is nearly the same.

**Figure 12:**
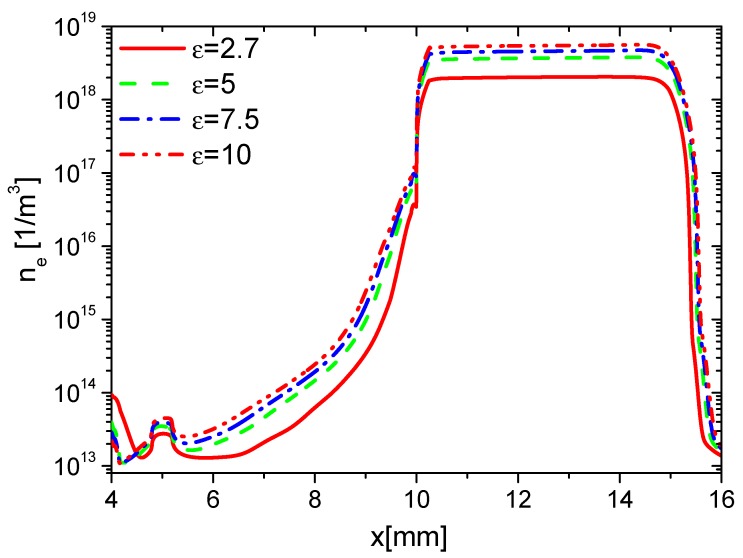
Effect of ε on the distributions of $n_{e}$ at 35 ns in the fall phase.

**Figure 13:**
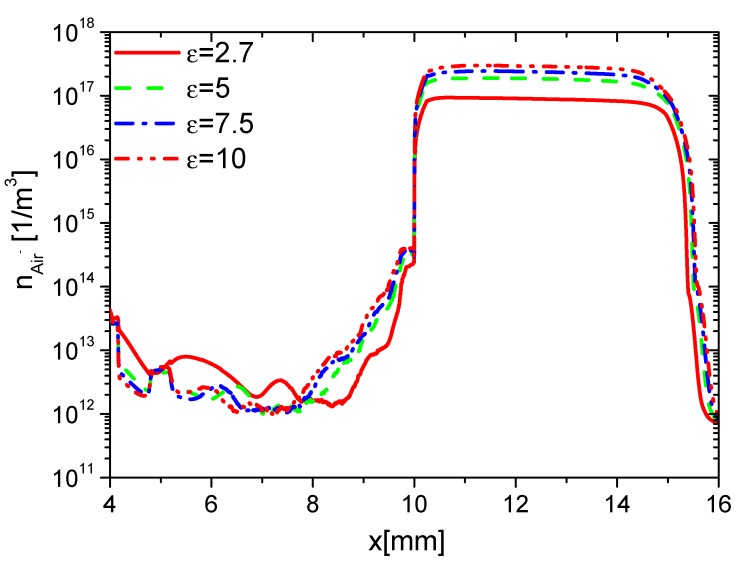
Effect of ε on the distributions of $n_{Air^{-}}$ at 35 ns in the fall phase.

**Figure 14:**
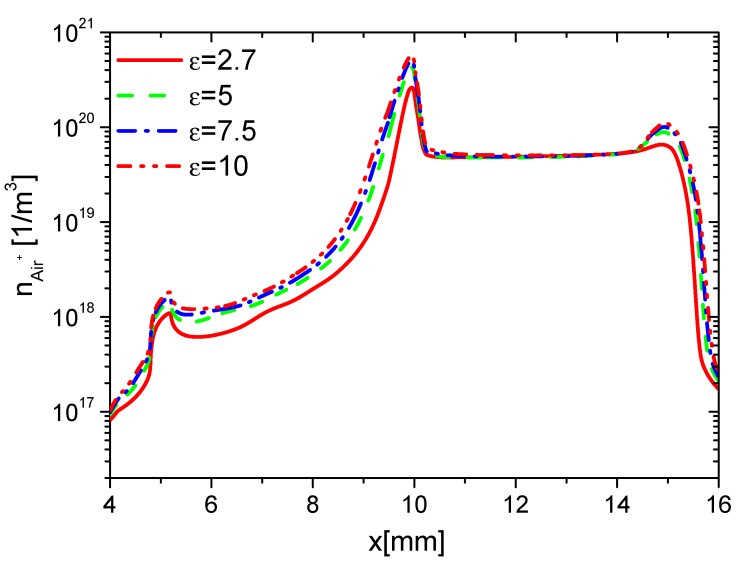
Effect of ε on the distributions of $n_{Air^{+}}$ at 35 ns in the fall phase.

**Figure 15:**
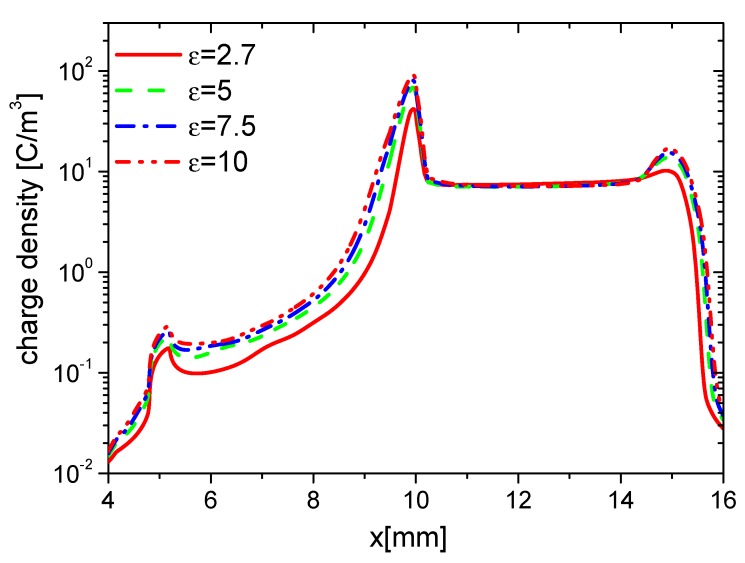
Effect of ε on the distributions of the wall charge density at 35 ns in the fall phase.

The densities of $n_{e}$, $n_{Air^{-}}$, $n_{Air^{+}}$, and charge on the wall surface in the fall phase at 35 ns are studied and shown in Figure 12, Figure 13, Figure 14 and Figure 15, respectively. $n_{e}$ and $n_{Air^{-}}$ increase in the whole wall surface with increasing ε because of the enhanced $\left|\overset{⇀}{\;E}\right|$. $n_{Air^{+}}$ also increases on the exposed electrode surface. However, it shows no obvious change on the dielectric surface. This is because $Air^{+}$ moves from the dielectric barrier to the exposed metal electrode, and the streamer mainly develops in the surface of the exposed electrode. The charge densities on the dielectric surface have no obvious difference, but they increase with increasing ε. This is also due to the streamer development along the exposed electrode surface in the fall phase.

According to the past numerical investigations [14,45,46], the surface charge density on the dielectric barrier surface of a DBD actuator increases with ε, where the peak charge density increases from 30 nC/cm${}^{2}$ to 135 nC/cm${}^{2}$ with ε varying from 2.5 to 10. The trend in the past studies is the same as the results in the present investigation.

#### 3.1.3. Streamer Morphology

In the present high pressure discharge condition, streamers form during the rise and fall phases, the streamer development, and the streamer morphology are similar to the ones in the previous paper [23]. The streamer propagation speed *V*, plasma sheath thickness *h*, and the maximum streamer length *L* in all the cases are shown in Table 2.

**Figure 16:**
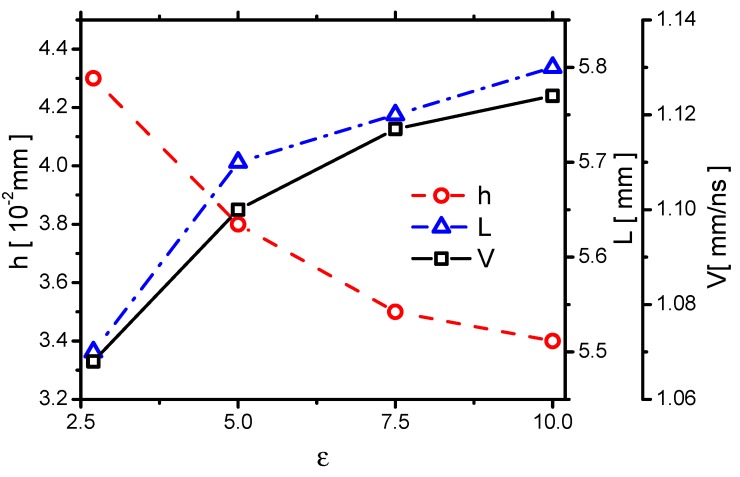
The effects of ε on the streamer propagation speed *V*, the plasma sheath thickness *h*, and the maximum streamer length *L* in Case 1 to Case 4.

First, the effect of ε on the streamer propagation speed *V* is investigated. *V* in Case 1 to Case 4 increases with increasing ε, as shown in Figure 16. To explain the result, a one-dimensional self-similar analytic solution for a streamer propagation in a quasi-two-dimensional domain [47] is considered, where *V* can be scaled by the peak electric field $E^{*}$ at the streamer head as $V∼e^{E^{*}}$. Because the electric field strength increases with increasing ε, *V* increases with increasing ε.

**Figure 17:**
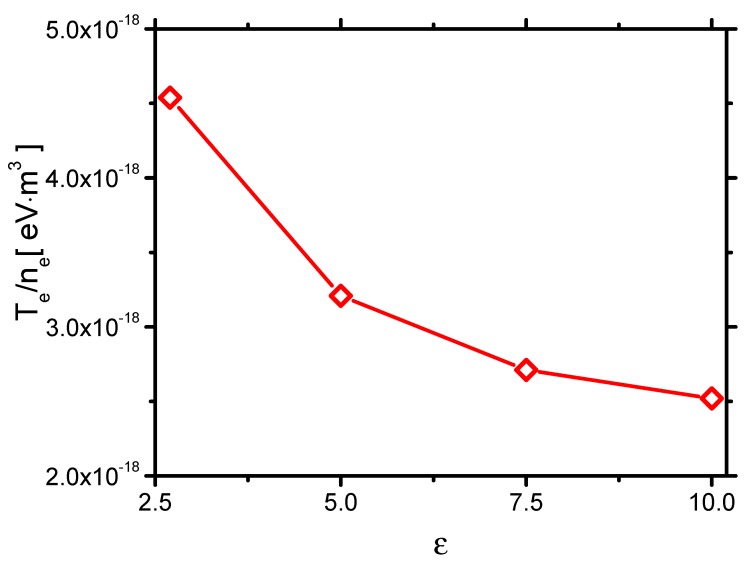
The effects of ε on $\frac{T_{e}}{n_{e}}$ in Case 1 to Case 4.

Second, the effect of ε on the plasma sheath thickness *h* is shown in Figure 16. *h* in Case 1 to Case 4 decreases with increasing ε. Theoretically, Franklin et al. [48] found that *h* can be related to the Debye length λD=εkBTeεkBTee2nee2ne12 as h∼λD45. The average electron temperature per unit electron density TeTenene is calculated from Figure 2, as shown in Figure 17. It decreases with increasing ε, which roughly follows the same trend as *h*.

Finally, the effect of ε on the maximum streamer length *L* is also shown in Figure 16. *L* in Case 1 to Case 4 increases with increasing ε. When ε increases by 3.7 times, from 2.7 to 10, *L* increases from $5.5\times 10^{-2}$ mm to $5.8\times 10^{-2}$ mm, by a factor 5%. It can be considered that *L* depends very weakly on ε; it is mainly determined by the length of the grounded electrode.

According to the previous studies [11], the streamer thickness increases significantly with an increase in ε, by a factor of 5 as ε changes from 4 to 80, which correlates with the results in the present study. In a recent experimental study and analytical predictions [14], the maximum streamer length decreases with growing ε in the range of 7–35. Because the streamer length in the experiment [14] is not limited by the electrode, the trend is different from the results in the present study.

The spatial distribution of gas heating is very crucial, and it is roughly determined by the streamer morphology [43]. When ε increases from 2.7 to 10 by 3.7 times, *V* increases from 1.068 to 1.124 by 5%, *h* decreases from 43 μm to 34 μm by 21%, and *L* increases by 5%. Therefore, the spatial distribution of gas heating is not much affected by ε.

#### 3.1.4. Discharge Energy and Gas Heating

**Figure 18:**
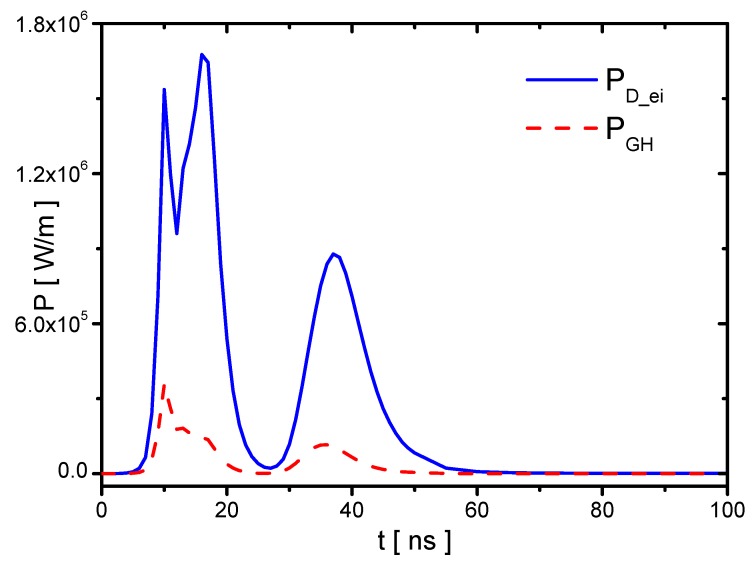
The powers of discharge energy $P_{D\_ei}$ and the fast gas heating $P_{GH}$ in Case 1.

**Figure 19:**
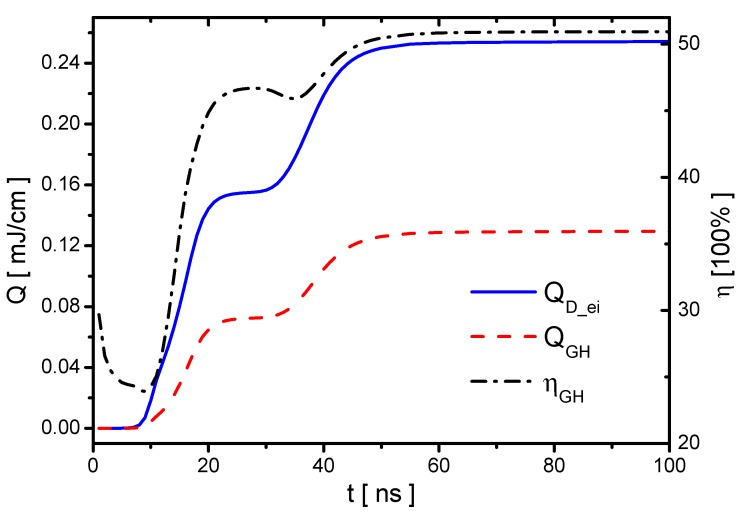
The discharge energy $Q_{D\_ei}$, the fast gas heating $Q_{GH}$ and the gas-heating efficiency $\eta_{GH}$ in Case 1.

The discharge energy and gas heating in the Case 1 are studied, and $P_{D\_ei}$ and $P_{GH}$ are shown in Figure 18. The instantaneous power $P_{D\_ei}$ has two peaks in the rise phase following the discharge current; however, $P_{GH}$ has only one peak mainly during the streamer development. Although the ionization occurs during the re-ignition in the plasma area covered previously by the streamer, the densities of electronic excited species and vibrational excited species cannot have much increments because they are already high in the streamer. Therefore, there is no obvious gas heating in the re-ignition process in the voltage-rise phase. In the voltage-fall phase, $P_{D\_ei}$ and $P_{GH}$ both have a peak because of the streamer in the reverse discharge. The energies of the discharge energy $Q_{D\_ei}$, gas heating $Q_{GH}$, and the corresponding gas heating efficiency $\eta_{GH}$ are shown in Figure 19. The increments of the discharge energy and gas heating with increasing ε in the rise and fall phase are nearly equal.

**Figure 20:**
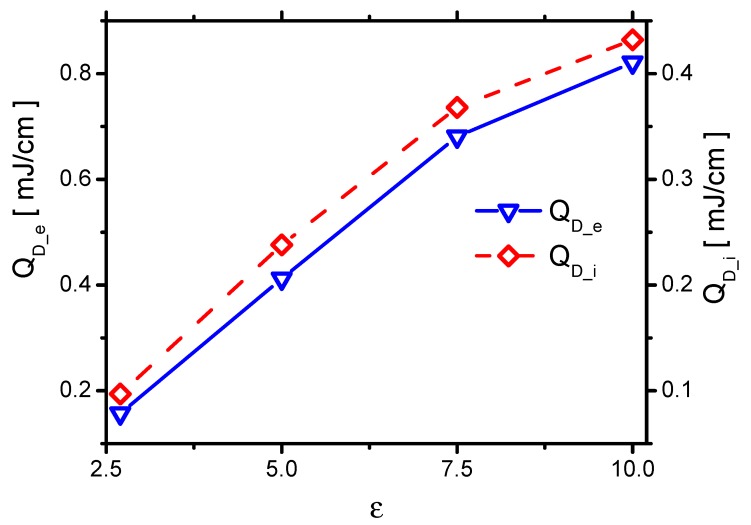
The effects of ε on the electrons’ energy $Q_{D\_e}$ and the ion’s energy $Q_{D\_i}$ in Case 1 to Case 4.

First, in order to know the effects of ε on $Q_{D\_ei}$, the effects of ε on $Q_{D\_e}$ and $Q_{D\_i}$ are studied, as shown in Figure 20. $Q_{D\_e}$ and $Q_{D\_i}$ increase with increasing ε. Theoretically, $Q_{D\_e}$ and $Q_{D\_i}$ originate from the movements of the electrons and the ions in the electric field. The products of $\left|\overset{⇀}{\;E}\right|$ and the mean-free path (λ), $\left|\overset{⇀}{\;E}\right|\lambda$, of the electrons and the ions are directly proportional to $Q_{D\_e}$ and $Q_{D\_i}$ [49]. $\left|\overset{⇀}{\;E}\right|$ increases with increasing ε. Kegel et al. [50] found that $\lambda ={\left(N\sigma \right)}^{-1}$, where σ and *N* are the cross-section of the collisions and the gas-number density, respectively. Because σ of ions depends on the gas temperature, which is independent of ε, $\left|\overset{⇀}{\;E}\right|\lambda$ of ions increases with $\left|\overset{⇀}{\;E}\right|$. Therefore, $Q_{D\_i}$ increases with increasing ε, which nearly follows the same trend as $\left|\overset{⇀}{\;E}\right|$. Due to the same reason, Eλ of electrons increases with increasing ε. $Q_{D\_e}$ increases with increasing ε, which also roughly follows the same trend of $\left|\overset{⇀}{\;E}\right|$.

**Figure 21:**
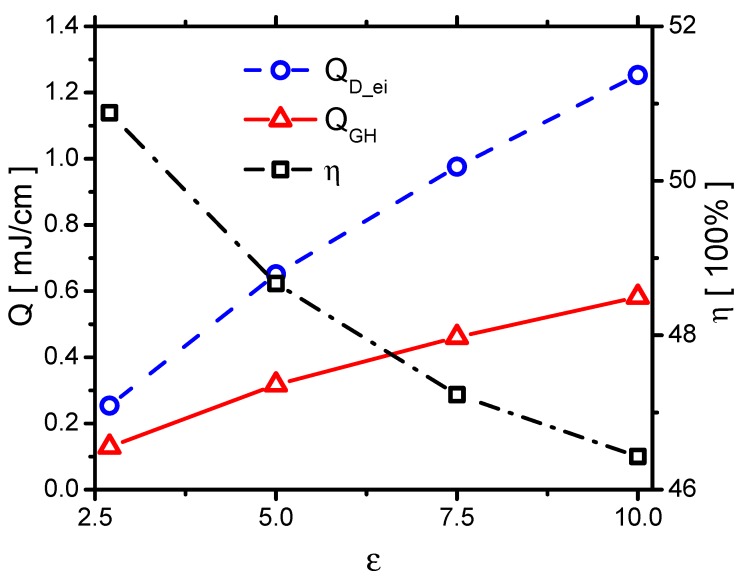
The effects of ε on the discharge energy $Q_{D\_ei}$, the gas heating $Q_{GH}$ and its efficiency $\eta_{GH}$ in Case 1 to Case 4.

The effects of $Q_{D\_ei}$ and $Q_{GH}$ are consequently concluded. They both increase with increasing ε, as shown in Figure 21. When ε increases from 2.7 to 10.0, $Q_{D\_e}$ increases from 0.16 mJ/cm to 0.82 mJ/cm by around 5 times. However, $Q_{D\_i}$ increases from 0.10 mJ/cm to 0.43 mJ/cm by 4.3 times. $Q_{D\_e}$ increases faster than the one of $Q_{D\_i}$. Because the ions transfer energy to the fast gas heating more efficiently than the electrons do, $\eta_{GH}$ decreases with increasing ε, as shown in Figure 21.

According to a previous experimental study conducted by Correale et al. [13], the total discharge energy changes by using different dielectric materials, and the discharge energy tends to increase with increasing ε. In addition, the size of the heated up area in the discharge, which can be associated with the magnitude of gas heating, roughly increases with ε. Therefore, the effects on the discharge energy and gas heating correlate with previous investigations.

The gas heating energy is very improtant. When ε increases from 2.7 to 10 by 3.7 times, $Q_{GH}$ increases from 0.129 mJ/cm to 0.582 mJ/cm by 4.5 times. Even the spatial distribution of the gas heating is much less affected by ε, the gas heating energy can be largely changed by ε. Therefore, varying the dielectric constant is a promising method to improve the performance of the actuator.

### 3.2. Effects of Dielectric Barrier Thickness

The thickness ($t_{d}$) of the dielectric barrier is another key parameter. In this section, the effect of $t_{d}$ is studied systemically.

#### 3.2.1. Plasma Characteristics and Current

**Figure 22:**
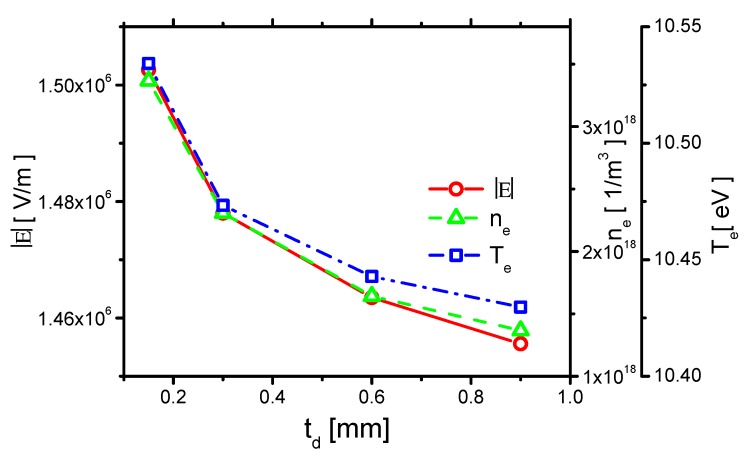
Effects of the thickness of the dielectric barrier $t_{d}$ on $\left|\overset{⇀}{\;E}\right|$, electron density $n_{e}$, and electron temperature $T_{e}$.

The effects of $t_{d}$ on the average $\left|\overset{⇀}{\;E}\right|$, $n_{e}$, and $T_{e}$ are shown in Figure 22, as the ones in Section 3.1.1.

$\left|\overset{⇀}{\;E}\right|$ decreases with increasing $t_{d}$. This is because a thinner dielectric barrier results in a lower capacitance, which is almost equal to increasing ε. As a result, the electric field strength increases with decreasing $t_{d}$. The electron density and electron temperature also increase with decreasing $t_{d}$.

**Figure 23:**
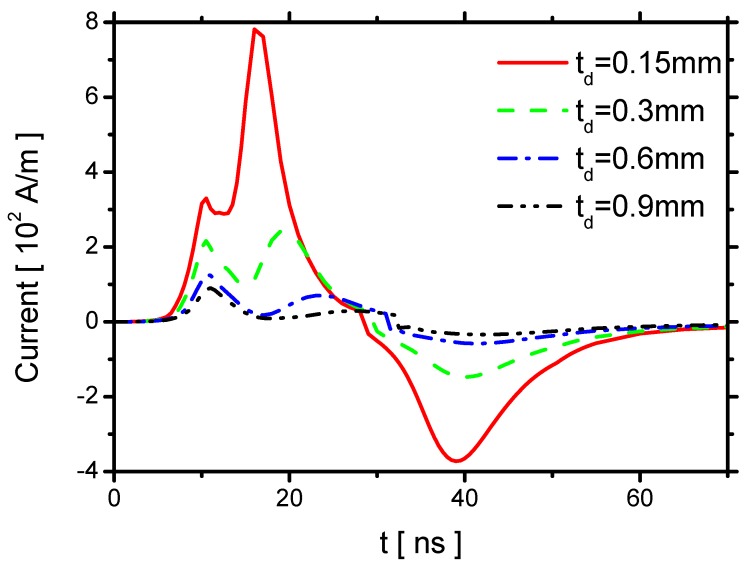
Effects of $t_{d}$ on the current.

The effect of $t_{d}$ on the current is shown in Figure 23. The two peaks of the current in the rise phase both decrease with increasing $t_{d}$. The second peak is negligible when $t_{d}=0.9$ mm. Because the streamer and the displacement current are reduced by decreasing $\left|\overset{⇀}{\;E}\right|$, the first peak decreases. Because the second peak is primarily determined by the capacitance of the dielectric barrier, the second peak is weaker when $t_{d}$ becomes larger. The currents in the reverse discharge in the fall phase also decrease with increasing $t_{d}$. According to the previous numerical study [11], for positive polarity discharge, the electric current decreases noticeably from 12∼25 A/cm to 3∼6 A/cm, when the dielectric thickness increases from 1 mm to 5 mm. The trend is the same as the ones in the present study.

#### 3.2.2. Surface Charge

**Figure 24:**
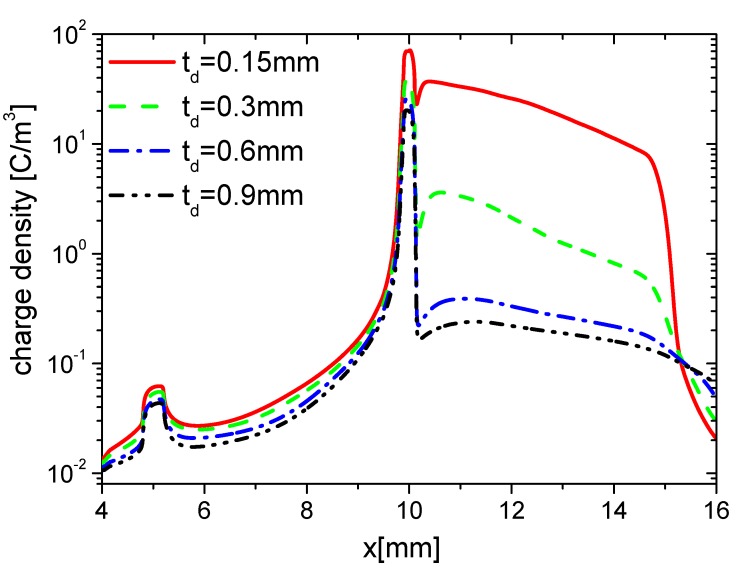
Effect of $t_{d}$ on the distributions of the wall charge density at 15 ns in the rise phase.

Because increasing $t_{d}$ results in the decrease of the capacitance of the dielectric barrier, the changing is equal to the decreasing of ε. It can be affirmed that the variation of surface charge with increasing $t_{d}$ is nearly the same as the one with decreasing ε. Here, effects of $t_{d}$ on the wall charge density are investigated in the rise and fall phases, respectively, as the ones in Section 3.1.2. The distributions of wall charge density at time 15 ns in the rise phase are shown in Figure 24. The charge density on the dielectric surface decreases, but they have no obvious differences on the exposed electrode surface, which is similar to the trends of ε in Figure 11. This is because $\left|\overset{⇀}{\;E}\right|$ decreases with increasing $t_{d}$; less charge is accumulated on the dielectric barrier surface in the rise phase.

**Figure 25:**
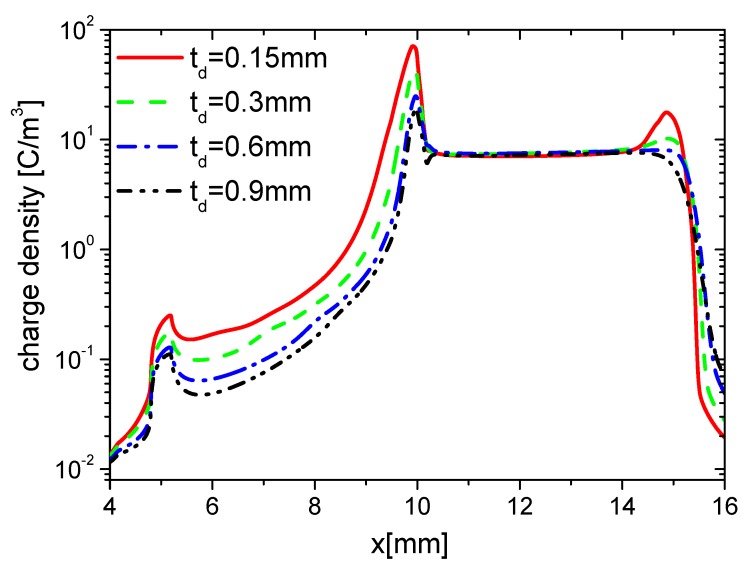
Effect of $t_{d}$ on the distributions of the wall charge density at 35 ns in the fall phase.

The distributions of wall charge density at time 35 ns in the fall phase are shown in Figure 25. The charge densities on the dielectric surface have no obvious differences, but they decrease with increasing $t_{d}$, which is also similar to the trend of ε in Figure 15. This is also due to the reduction of $\left|\overset{⇀}{\;E}\right|$ with increasing $t_{d}$, which weakens the charge in the streamer along the exposed electrode surface in the fall phase.

Because the changes of $t_{d}$ and ε are projected to the change of capacitance, the effects of $t_{d}$ on the distributions of the electrons and ions on the wall surface are nearly the same as the ones of ε in Section 3.1.2. They are not given in detail here.

#### 3.2.3. Streamer Morphology

**Figure 26:**
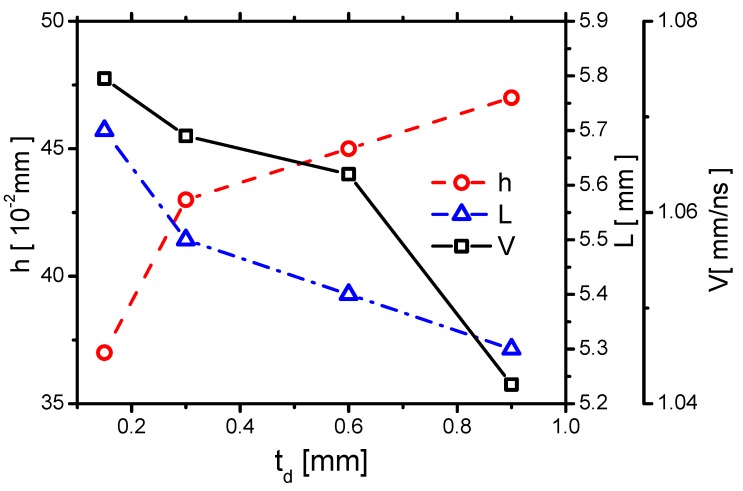
The effects of $t_{d}$ on the streamer propagation speed *V*, the plasma sheath thickness *h*, and the maximum streamer length *L* in Case 1 and Cases 5–7.

First, the effect of $t_{d}$ on *V* is shown in Figure 26, where *V* decreases with increasing $t_{d}$. The trend is nearly similar to the cases in decreasing ε in Figure 16. It can be explained by using the one-dimensional self-similar analytic solution for a streamer propagating in a quasi-two-dimensional domain [47] in Section 3.1.3, where *V* is determined by the peak electric field $E^{*}$ at the streamer head. Because the electric field strength decreases with increasing $t_{d}$, *V* decreases with increasing $t_{d}$.

**Figure 27:**
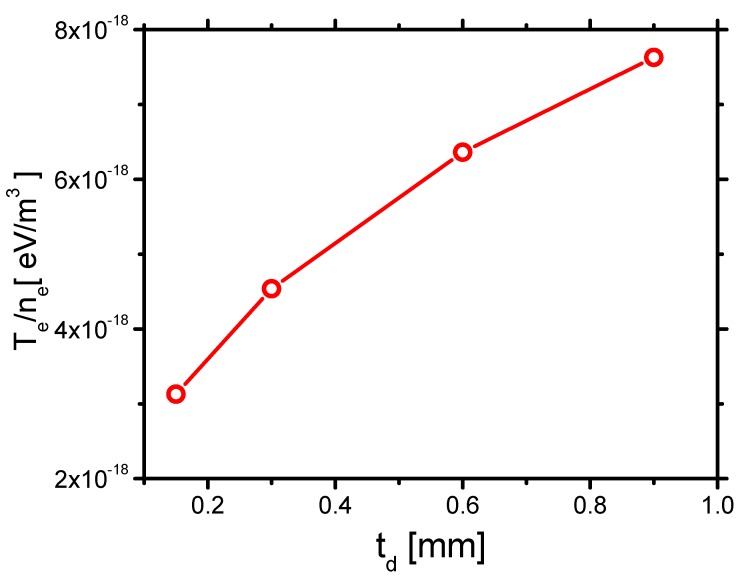
The effects of $t_{d}$ on the average electron temperature per unit electron density $\frac{T_{e}}{n_{e}}$ in Case 1 and Cases 5–7.

Second, the effect of $t_{d}$ on *h* is shown in Figure 26. *h* increases with increasing $t_{d}$, which roughly follows the same trend as decreasing ε, as shown in Figure 16. Considering the relation of *h* and the Debye length h∼λD45 [48] in Section 3.1.3, and the average electron temperature per unit electron density TeTenene increasing with increasing $t_{d}$ as shown in Figure 27, the trend of *h* is explained.

Finally, the effect of $t_{d}$ on *L* is also shown in Figure 16, where *L* decreases with increasing ε. However, *L* also depends very weakly on $t_{d}$ because it decreases from $5.7\times 10^{-2}$ mm to $5.3\times 10^{-2}$ mm, by a factor 7%, when $t_{d}$ increases by 6 times, from 0.15 mm to 0.9 mm. *L* is also mainly determined by the length of the grounded electrode 5 mm.

According to the previous study [11], the streamer thickness increases with an increase in $t_{d}$, which correlates with the results in the present study. When $t_{d}$ increases from 0.15 mm to 9 mm, by 6 times, *V* decreases from 1.074 to 1.042 by 2.9%, *h* increases from 37 μm to 47 μm by 27%, and *L* decreases by 7%. Therefore, the spatial distribution of gas heating, which is determined by the streamer morphology, is not much affected by $t_{d}$.

#### 3.2.4. Discharge Energy and Gas Heating

**Figure 28:**
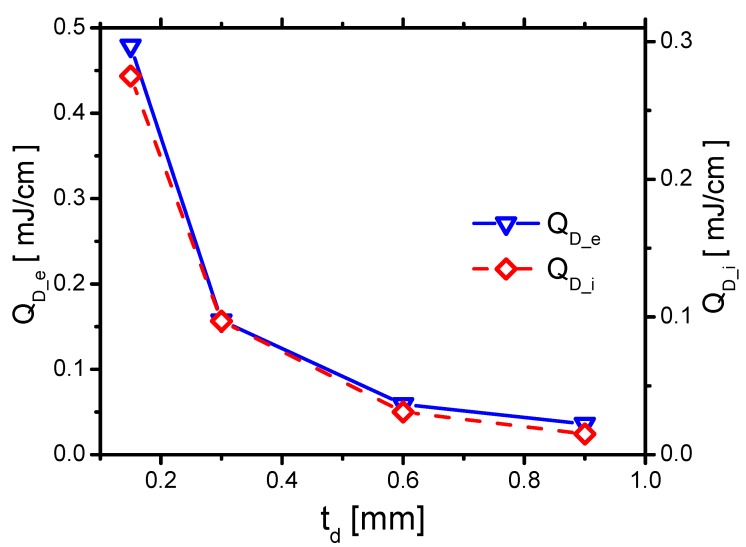
The effects of $t_{d}$ on the electrons’ energy $Q_{D\_e}$ and the ion’s energy $Q_{D\_i}$ in Case 1 and Cases 5–7.

First, to know the effects of $t_{d}$ on $Q_{D\_ei}$, the effects of $t_{d}$ on $Q_{D\_e}$ and $Q_{D\_i}$ are analyzed and shown in Figure 28. $Q_{D\_e}$ and $Q_{D\_i}$ decrease with increasing $t_{d}$, which are similar to the ones in decreasing ε. This is because $Q_{D\_e}$ and $Q_{D\_i}$ are all affected by the products of $\left|\overset{⇀}{\;E}\right|$ and the mean-free path (λ), $\left|\overset{⇀}{\;E}\right|\lambda$. $\left|\overset{⇀}{\;E}\right|$ decreases with increasing $t_{d}$. λ of electrons and ions are all nearly unaffected by $t_{d}$. Therefore, the trends of $Q_{D\_e}$ and $Q_{D\_i}$ roughly follow the same trend as $\left|\overset{⇀}{\;E}\right|$.

**Figure 29:**
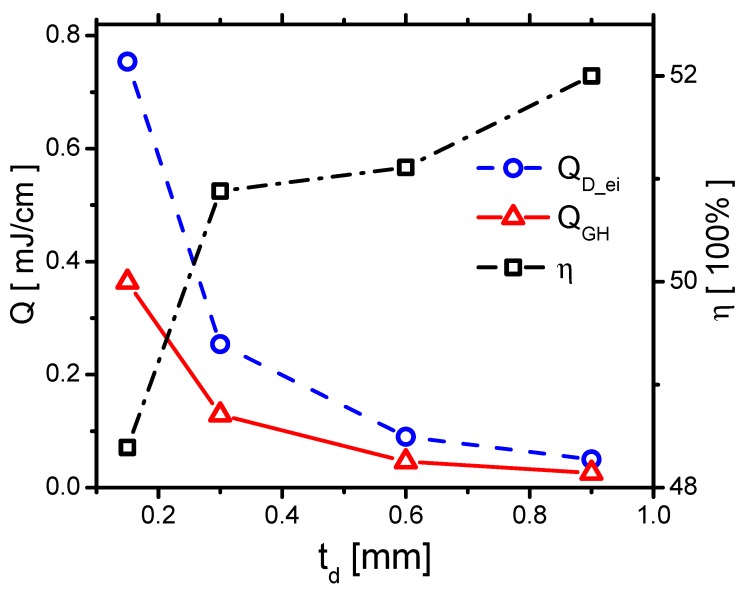
The effects of $t_{d}$ on the discharge energy $Q_{D\_ei}$, the gas heating $Q_{GH}$ and its efficiency $\eta_{GH}$ in Case 1 and Cases 5–7.

The effects of $Q_{D\_ei}$ and $Q_{GH}$ are consequently concluded. They both decrease with increasing $t_{d}$, as shown in Figure 29. When $t_{d}$ increases from 0.15 mm to 0.9 mm, $Q_{D\_e}$ decreases from 0.479 mJ/cm to 0.036 mJ/cm by around 13 times. However, $Q_{D\_i}$ decreases from 0.275 mJ/cm to 0.015 mJ/cm by 18 times. $Q_{D\_e}$ decreases faster than the one of $Q_{D\_i}$. Because the ions transfer energy to the fast gas heating more efficiently than the electrons do, $\eta_{GH}$ increases with increasing $t_{d}$, as shown in Figure 29.

According to the previous experimental studies [15], the discharge energy per pulse decreases with $t_{d}$ increases from 120 μm to 360 μm. The same conclusions can also be found in another experiment investigation [16], where the discharge energy and power decrease with increasing $t_{d}$. In addition, Correale et al. [13] found that, for a thicker dielectric barrier, less gas heating energy is actually deposited in the discharge volume. Therefore, the effects obtained on the discharge energy and gas heating correlate with previous investigations.

When $t_{d}$ decreases from 0.9 mm to 0.15 mm, by 6 times, $Q_{GH}$ increases from 0.036 mJ/cm to 0.479 mJ/cm by 13 times. Although the spatial distribution of the gas heating is much less affected by $t_{d}$, the gas heating energy can be considerably enhanced by decreasing $t_{d}$. Therefore, changing the thickness of the dielectric barrier is also a promising method for improving the performance of the actuator.

## 4. Conclusions

The effects of dielectric constant and dielectric barrier thickness of the dielectric barrier on the discharge characteristics of an asymmetric assembled NS-DBD have been studied by a three-equation drift–diffusion model. An air-chemistry model containing four species and four reactions is adopted in the simulation. LMEA is used to obtain the electron–impact reaction-rate coefficients and the electron–transport coefficients.

When the dielectric constant increases from 2.7 to 10, the streamer propagation speed (*V*), the maximum streamer length (*L*), the discharge energy ($Q_{D\_ei}$), and the gas heating ($Q_{GH}$) of a pulse increase, but the plasma sheath thickness (*h*) and the fast gas heating efficiency η decrease. *V* increases from 1.068 to 1.124 by 5%. *L* increases from $5.5\times 10^{-2}$ mm to $5.8\times 10^{-2}$ mm by 5%. *h* decreases from 43 μm to 34 μm by 21%. $Q_{D\_ei}$ increases from 0.254 mJ/cm to 1.253 mJ/cm by 4.9 times. $Q_{GH}$ increases from 0.129 mJ/cm to 0.582 mJ/cm by 4.5 times. η varies from 50.88% to 46.43%. The charge densities on the surface of the dielectric barrier and the exposed electrode increase with increasing of the dielectric constant.

When the dielectric barrier thickness increases from 0.15 mm to 0.9 mm, *V*, *L*, $Q_{D\_ei}$, and $Q_{GH}$ of a pulse decrease, but *h* and η increase. *V* decreases from 1.074 to 1.042 by 2.9%, and *L* decreases from $5.7\times 10^{-2}$ mm to $5.3\times 10^{-2}$ mm by 7%. *h* increases from 37 μm to 47 μm by 27%. $Q_{D\_ei}$ increases from 0.754 mJ/cm to 0.050 mJ/cm by 15 times. $Q_{GH}$ increases from 0.036 mJ/cm to 0.479 mJ/cm by 13 times. η changes from 48.39% to 52.00%. The charge densities on the dielectric barrier and exposed electrode decrease with increasing of the dielectric barrier thickness.

Either increasing the dielectric constant or decreasing the dielectric barrier thickness results in the increase of the capacitance of the dielectric barrier, which further affects the discharge characteristics. Although the streamer morphology is not much affected by the dielectric constant and the thickness, the gas heating energy can be largely enhanced by changing the dielectric constant and the thickness of the dielectric barrier of an NS-DBD.

## Figures and Tables

**Table 1:** Reaction mechanism. Ei denotes the electronic ionization. Att denotes the attachment. Re_ei and Re_ii denote the recombination of electron–ion and ion–ion, respectively.

No.	Reaction	Reaction Type	Rate Expression
(1)	$Air+e\to Air^{+}+2e$	Ei	α
(2)	$Air+e\to Air^{-}$	Att	η
(3)	$Air^{+}+e\to Air$	Re_ei	$2\times 10^{-7}$
(4)	$Air^{+}+Air^{-}\to 2Air$	Re_ii	2×10−7×300300TT

**Table 2:** List of the simulation cases and the results. *L*, *h* and *V* are the maximum streamer length, the plasma sheath thickness and the streamer propagation speed, respectively. $Q_{D\_ei}$, $Q_{GH}$, and $\eta_{GH}$ are the total discharge energy, the gas heating, and the gas-heating efficiency of a pulse, respectively.

Case	ε	$t_{d}$	L	h	V	$Q_{D\_\mathrm{ei}}$	$Q_{\mathrm{GH}}$	$\eta_{\mathrm{GH}}$
		[mm]	[mm]	[$10^{-2}$ mm]	[mm/ns]	[mJ/cm]	[mJ/cm]	[100%]
1	2.7	0.3	5.5	4.3	1.068	0.254	0.129	50.88
2	5.0	0.3	5.7	4.0	1.100	0.651	0.317	48.67
3	7.5	0.3	5.75	3.5	1.117	0.976	0.461	47.23
4	10.0	0.3	5.8	3.4	1.124	1.253	0.582	46.43
5	2.7	0.15	5.7	3.7	1.074	0.754	0.364	48.39
6	2.7	0.6	5.4	4.5	1.064	0.090	0.046	51.11
7	2.7	0.9	5.3	4.7	1.042	0.050	0.026	52.00
